# Chloroplast incorporation and long-term photosynthetic performance through the life cycle in laboratory cultures of *Elysia timida* (Sacoglossa, Heterobranchia)

**DOI:** 10.1186/1742-9994-11-5

**Published:** 2014-01-16

**Authors:** Valerie Schmitt, Katharina Händeler, Susanne Gunkel, Marie-Line Escande, Diedrik Menzel, Sven B Gould, William F Martin, Heike Wägele

**Affiliations:** 1 Zoologisches Forschungsmuseum Alexander Koenig, Adenauerallee 160, 53113 Bonn, Germany; 2 Institut für Molekulare Evolution, Heinrich-Heine-Universität Düsseldorf, Universitätsstr. 1, 40225 Düsseldorf, Germany; 3 Observatoire Océanologique, 66651 Banyuls-sur-mer, France; 4 Institut für Zelluläre und Molekulare Botanik, Zellbiologie der Pflanzen, Rheinische Friedrich-Wilhelms-Universität Bonn, Kirschallee 1, 53115 Bonn, Germany

**Keywords:** Endosymbiosis, Chloroplasts, Kleptoplasty, Photosynthetic sea slug, Solar powered sea slug, Sacoglossa, *Elysia*, Model organism

## Abstract

**Introduction:**

The Mediterranean sacoglossan *Elysia timida* is one of the few sea slug species with the ability to sequester chloroplasts from its food algae and to subsequently store them in a functional state in the digestive gland cells for more than a month, during which time the plastids retain high photosynthetic activity (= long-term retention). Adult *E. timida* have been described to feed on the unicellular alga *Acetabularia acetabulum* in their natural environment. The suitability of *E. timida* as a laboratory model culture system including its food source was studied.

**Results:**

In contrast to the literature reporting that juvenile *E. timida* feed on *Cladophora dalmatica* first, and later on switch to the adult diet *A. acetabulum*, the juveniles in this study fed directly on *A. acetabulum* (young, non-calcified stalks); they did not feed on the various *Cladophora spp.* (collected from the sea or laboratory culture) offered. This could possibly hint to cryptic speciation with no clear morphological differences, but incipient ecological differentiation. Transmission electron microscopy of chloroplasts from *A. acetabulum* after initial intake by juvenile *E. timida* showed different states of degradation — in conglomerations or singularly — and fragments of phagosome membranes, but differed from kleptoplast images of *C. dalmatica* in juvenile *E. timida* from the literature. Based on the finding that the whole life cycle of *E. timida* can be completed with *A. acetabulum* as the sole food source, a laboratory culture system was established. An experiment with PAM-fluorometry showed that cultured *E. timida* are also able to store chloroplasts in long-term retention from *Acetabularia peniculus*, which stems from the Indo-Pacific and is not abundant in the natural environment of *E. timida*. Variations between three experiment groups indicated potential influences of temperature on photosynthetic capacities.

**Conclusions:**

*E. timida* is a viable laboratory model system to study photosynthesis in incorporated chloroplasts (kleptoplasts). Capacities of chloroplast incorporation in *E. timida* were investigated in a closed laboratory culture system with two different chloroplast donors and over extended time periods about threefold longer than previously reported.

## Introduction

The phenomenon of “long-term retention” of functional chloroplasts from food algae with ongoing photosynthesis inside the slugs’ cells over longer time periods than a month only occurs in very few sea slug species [1-5]. Among animals, such kleptoplasty is only known for sacoglossan sea slugs [4-6]. Thus, these plastid bearing marine slugs are interesting in their own right, but they can further act as model organisms to study a special kind of ‘‘symbiosis’’. With few exceptions that we discuss below, most sacoglossan studies were performed on individuals collected from the sea, which implies that the history of the animals before collection, that is for example their age, repertoire of algae they have fed on or light conditions they have experienced is unknown. Transmission electron microscopy (TEM) and, more recently, molecular analyses provided insights into their food spectrum [4,7-15], but overall data is still sparse. Rumpho and coworkers kept the long-term retention species *Elysia chlorotica* Gould, 1870 [16] successfully in a laboratory culture system and characterized the entire life span of approximately ten months for the first time [3]. In a new study they report lab-reared cultures of regularly fed individuals they kept for more than two years, and without observing ‘annual mortality’ that earlier reports had documented [17]. Within their laboratory culture system they found that juveniles needed to feed on *Vaucheria litorea* (Agardh, 1823) [18] for at least seven days to establish kleptoplasty [17]. Curtis and coworkers [12] raised slugs hatched from egg masses laid in the laboratory by adult sea slugs, which were originally collected from the sea and fed the offspring after metamorphosis, but only for a limited period of time for subsequent experiments.

The laboratory culture of sea slugs permits long-term studies under controlled conditions und with animals of known individual history. It allows developmental investigations and reduces the burden on natural sea slug populations [3,17]. Laboratory culture can foster research progress on kleptoplast maintenance in slugs, which is still poorly understood [1-4,19-27]. Several interesting evolutionary adaptations in relation to long-term retention of chloroplasts are described for *Elysia timida* (Risso, 1818) [28], including positive phototaxis and closing or opening their parapodial lobes to modulate light flux [29-32]. Further, a physiological photo-regulation mechanism in form of the xanthophyll cycle to increase the maintenance of its photosynthetic capacities was also postulated [30].

Here we report the captive breeding and culturing of *E. timida,* which provides the opportunity to use this slug as a novel model organism to study feeding behavior, chloroplast sequestration, long-term kleptoplast retention and kleptoplast photosynthesis throughout the slugs’ entire life cycle.

## Results

**Figure 1:**
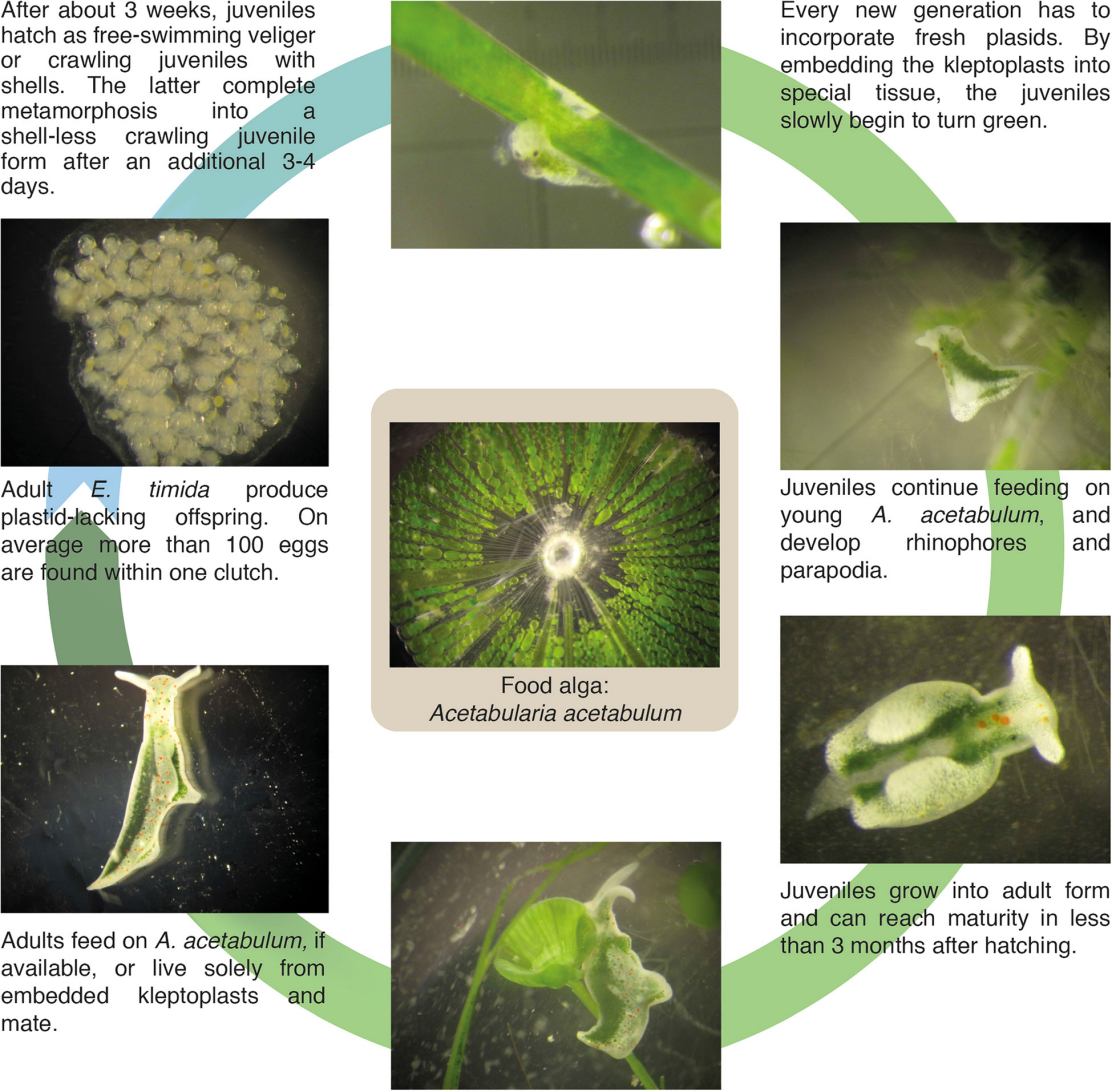
**Life cycle of *Elysia timida*.** The whole life cycle of *E. timida* can be completed by feeding on the food alga *Acetabularia acetabulum* shown in the centre.

*Elysia timida* individuals collected from the sea mated frequently under laboratory conditions and produced considerable amounts of egg masses (Table 1). The stability and orange coloration proved to be advantageous for culturing, as clutches could be recognized easily in the aquaria, removed and handled separately in petri dishes. Within short development periods of up to three weeks, the offspring developed in their egg capsules and hatched as free-swimming veliger or as crawling juveniles, with the larval shell still attached (Table 2). Final metamorphosis into shell-less, crawling juveniles took place within three to four days and without the presence of any algae. The whole development of freshly laid clutches into metamorphosed juveniles was completed within 24.8 ± 3.0 days on average in the example group of observed clutches in the marine laboratory Observatoire Océanologique Banyuls-sur-mer, and 20.0 ± 2.6 days in the example group of observed clutches later in the laboratory culturing system in Düsseldorf (Table 2). More than 100 eggs on average per single clutch were counted in an example group of 45 clutches in the laboratory culturing system (Table 3). In an exemplary clutch containing 215 eggs, the entire life cycle (Figure 1) was observed. After 106 days 122 individuals were still alive, translating into a survival rate of 57%. At this time point two new clutches were found in the glass bowl, indicating sexual maturity of the reared offspring.

**Table 1:** Reproduction under laboratory conditions

**Tank group**	**1**	**2**
N° individuals	92	87
Captivity period [days]	44	28
N° clutches	218	207
Average of clutches per day	4.95	7.39
Average of clutches per individual	2.37	2.38

Number of clutches in two different groups of adult *Elysia timida* kept at the marine laboratory OOB (Observatoire Océanologique Banyuls-sur-mer, France). The sea slugs were collected freshly from the sea and kept in two 5 1 tanks with aerated seawater and constant supply of *Acetabularia acetabulum* food algae.

**Table 2:** Development under laboratory conditions

	**Marine laboratory (OOB) n = 37, 16–22°C**		**Laboratory culturing system (IME) n = 17, 20–23°C**	
**Metamorphosis state**	**Mean**	**SD**	**Mean**	**SD**
Period since clutch deposition until hatching as veliger or juvenile with shell [days]	20.9	2.4	16.7	2.7
Metamorphosis from hatching into shell-less crawling juvenile [days]	3.8	1.0	3.3	1.9
Total development time from clutch deposition to shell-less crawling juvenile [days]	24.8	3.0	20.0	2.6

Time periods from egg laying to development of shell-less crawling juveniles of *Elysia timida* at the OOB (Oberservatoire Océanologique Banyuls-sur-mer, France) and IME (Institute for Molecular Evolution, Düsseldorf, Germany). SD standard deviation.

**Table 3:** Reproductive output under laboratory conditions

	**N° eggs per clutch**
Mean	108.36
SD	52.75
Minimum	19
Maximum	249

Average, minimum and maximum numbers of eggs in spawn of *Elysia timida* (counted in an example group of 45 clutches).

**Figure 2:**
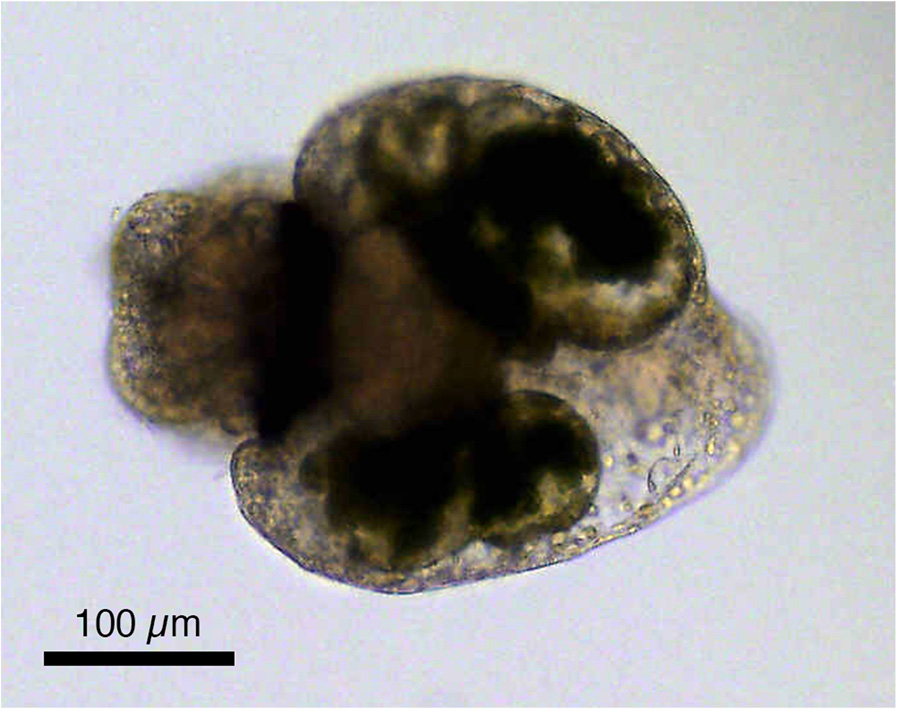
**Juvenile *Elysia timida *after the first uptake of *Acetabularia acetabulum *cytosol.** When transparent juveniles feed for the very first time on *A. acetabulum*, the cytosol of the algae including the plastids, fill up the digestive gland, thereby revealing its bilobed structure. The individual was fixed 2–3 hours after the first *A. acetabulum* meal, and about 5–6 days after hatching. The individual is oriented with its head to the left side, the digestive gland can be seen in the middle with the two balloon-like protrusions that are filled up and colored greenish by the plastids.

**Figure 3:**
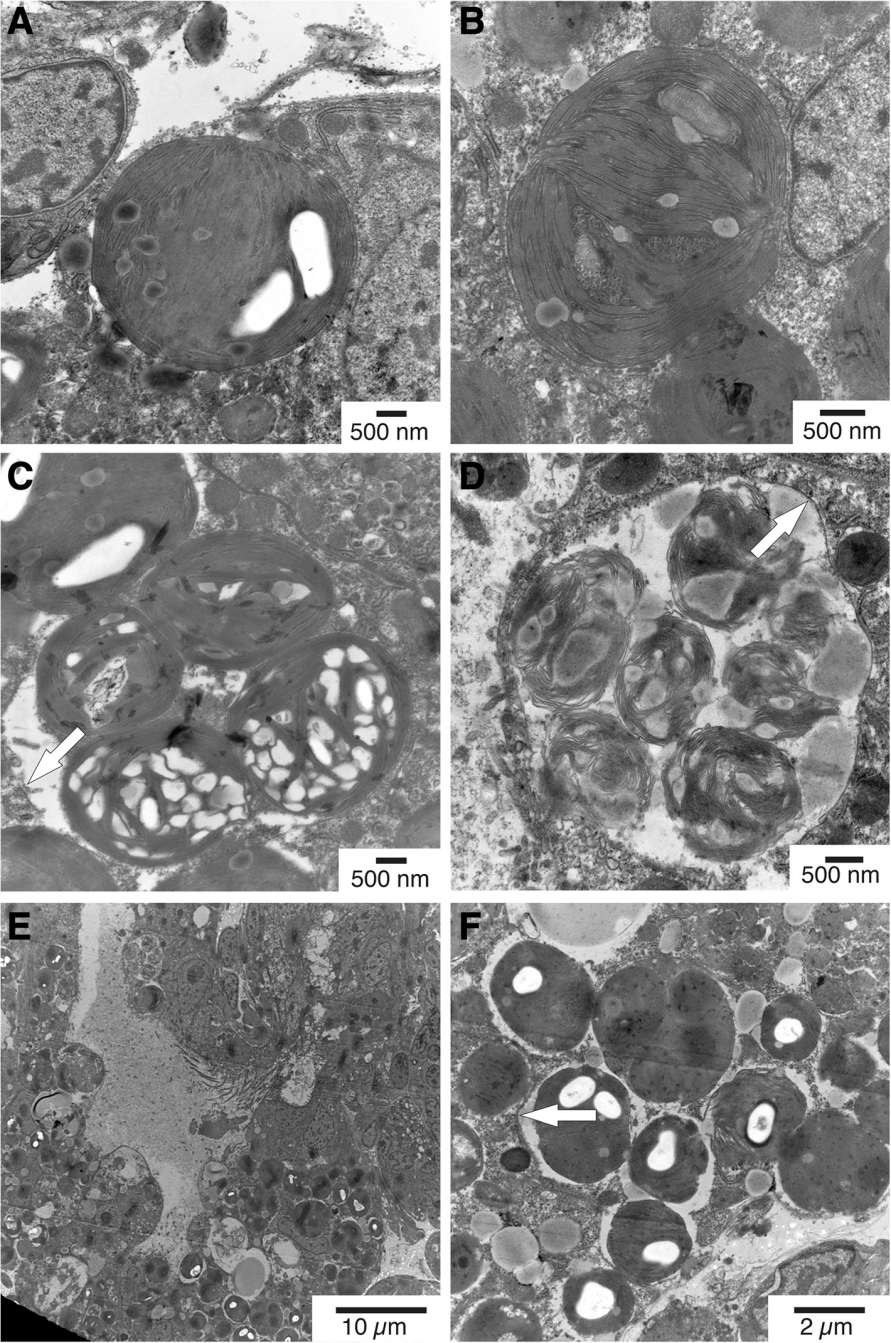
**Ultrastructural investigations.** Chloroplasts in digestive glandular cells in two different juvenile *Elysia timida* specimens fixed 2–3 h after the beginning of the very first feeding on *Acetabularia acetabulum ***(A,B,C)***.* Chloroplasts in different states of degradation in juvenile *E. timida* fixed 2 days after the beginning of the very first feeding on *A. acetabulum* and free food supply until fixation **(D,E,F)**. Around some degrading chloroplasts gaps are evident and some are enclosed in conglomerations. Fragments of phagosome membranes are highlighted by arrows.

Juvenile *E. timida* fed directly on the diet usually consumed by adult slugs, *A. acetabulum*, when young non-calcified stalks collected from the sea in the environment of adult slug populations were presented. In contrast to this, none of the hatched juveniles from more than 20 observed clutches fed on any *Cladophora spp.* sampled at the collection sites. This was confirmed through laboratory feeding trials with juveniles, which fed exclusively on *A. acetabulum* and not *C. dalmatica* or any of the other algae offered (Table 4). Based on these findings a closed laboratory culture system of *E. timida*, and which included simultaneous cultivation of *A. acetabulum*, was established (Figure 1). As plastids are not inherited by the offspring from the adult slugs, juveniles are transparent after hatching. Upon initial feeding on *A. acetabulum*, juveniles take up the green chloroplasts into the digestive gland revealing its bilateral structure (Figure 2; Additional file 1). The uptake of chloroplasts into the digestive glandular cells was documented by TEM (Figure 3) at two different time points: (1) juveniles were fixed 2–3 hours after feeding on *A. acetabulum* had commenced, and (2) a second group was preserved two days after permanent supply of *A. acetabulum*. In both cases chloroplasts in various states were found, in single and as aggregates. Juveniles that had been fixed 2–3 hours after their first chloroplasts meal and had been continuously feeding before fixation showed more single chloroplasts that still appeared intact than in a juvenile fixed two days after the beginning of feeding and constant food supply, in which more pronounced degradation was observed. Aggregates of chloroplasts surrounded by a phagosome membrane and with first signs of degradation were also visible in individuals fixed 2–3 hours after initiation of feeding (Figure 3C). While intact chloroplasts appeared to be embedded directly in the cytoplasm, chloroplasts in the process of degradation showed pronounced gaps between them and their surroundings and partially fragmented phagosome membranes. These gaps were more pronounced around aggregates of several chloroplasts (Figure 3A-F).

**Table 4:** **Algae consumption in juvenile *Elysia timida***

	**Consumed by juveniles**	**N° positive/total in two trial series**
*Acetabularia acetabulum*	yes	6/25 (n = 10 + 15)
*Acetabularia peniculus*	no	0/25 (n = 10 + 15)
*Cladophora dalmatica*	no	0/25 (n = 10 + 15)
*Cladophora rupestris*	no	0/25 (n = 10 + 15)

Feeding acceptance of juvenile *E. timida* in the laboratory culture system was tested. Two series with juveniles from two different clutches (40 individuals from the first clutch and 60 individuals from the second clutch) were performed and observed during 3 weeks. Feeding was determined by the green coloration through uptake of algal sap into the transparent juveniles.

**Figure 4:**
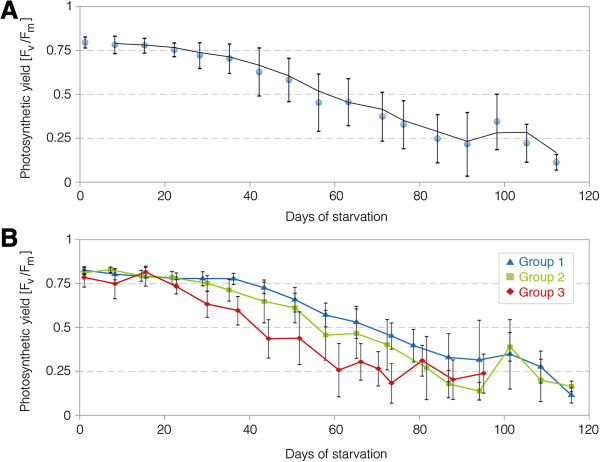
**Photosynthetic long-term performance of *Acetabularia acetabulum *plastids in *Elysia timida *during starvation. A)** Photosynthetic yields of PAM-measurements of all the 50 *E. timida* individuals included in the experiment. **B)** Photosynthetic yields of PAM-measurements, divided into the three different serial trial groups of *E. timida* individuals [Group1: start January 31st 2012, n = 24, temperature range: 19(or lower)-22°C; group 2: start March 13th 2012, n = 16, temperature range 20-22°C; group 3: start June 25th 2012, n = 10, temperature range: 20-24°C]. Regular interval temperature measurements started end of March to avoid overheating due to rising temperatures in spring and summer.

In order to examine if adult *E. timida* feed on other algae species and incorporate their chloroplasts, we designed a three-phased experiment (see material and methods for details). In total, 50 individuals from the laboratory culture kept on *A. acetabulum* were first starved until the photosynthetic yield values of Pulse Amplitude Modulated fluorometry (PAM)-measurements approached F_v_/F_m_ values of zero (Phase 1, Figure 4A). Three different groups with different temperature background were used (for details see below). Subsequently, a set of different algae was offered in individual feeding trials lasting one month (Phase 2). These individuals were then again subjected to starvation and photosynthetic activity documented through PAM measurements (Phase 3). For the total group of 50 individuals, yield values remained on a high level of photosynthetic activity of about 0.8 during more than a month of starvation, reflecting high levels of intact chloroplasts (Figure 4A). PAM values then slowly decreased indicating gradual degradation of more and more chloroplasts, until the green coloration of the slugs bleached after about 90 days (88,56 ± 22,64 days; range: 42–135 days; n = 46 surviving until bleached and approaching zero). F_v_/F_m_ values at this point approached zero on average.

The 50 individuals were investigated in three subsequent series during phase 1, with the first starting in winter, the second in spring and the third in summer (end of January, middle of March 2012 and end of June, respectively; Figure 4B). The first group of *E. timida* individuals (winter group; blue) was exposed to the overall coldest temperatures and revealed the best photosynthetic performance with yield values staying at high levels the longest. The spring series (Figure 4B; green) had the same maximum ambient temperature of 21.6°C measured as the first group, but was exposed on average to a slightly higher temperature. This group showed a slightly lower long-term photosynthetic activity than the winter group. The summer group (Figure 4B; red), which was exposed to the overall highest temperatures – especially during the first phase of the experiment – with highest measured values reaching 24.0°C, showed a faster decrease of photosynthetic activity (Figure 4B).

In total, 39 of the 50 specimens survived the first starvation period (Phase 1) and thus could be included into phase 2 of the experiment. Of these, nine specimens were fed on *A. acetabulum* and nine on *A. peniculus,* which led to an increase of photosynthetic yield values measured (Table 5). During the re-feeding phase (phase 2), individuals fed with *A. acetabulum* showed overall only slightly higher photosynthetic yield values of 0.76 ± 0.11 (range: 0.31-0.86), than those fed with *A. peniculus* (0.72 ± 0.09; range: 0.44-0.82). Like juveniles, the adults (15 individuals tested) did neither feed on *C. dalmatica* nor on other potential plastid donors (*V. litorea* and *C. verticillata)*, the food of other long-term retention sea slugs species (*E. chlorotica* and *E. clarki*, respectively; Table 5). These individuals did not show a photosynthetic yield recovery and often quickly died despite of being supplied with the test algae.

**Table 5:** **Algae consumption in adult *Elysia timida***

	**Fed on by adult *E. timida***	**N° trials n = 39**
*Acetabularia acetabulum*	yes	Positive: 9
		Negative: 2
		Unsure:1
*Acetabularia peniculus*	yes	Positive: 9
		Negative: 2
		Unsure:1
*Cladophora dalmatica*	no	3 (all negative)
*Vaucheria litorea*	no	8 (all negative)
*Caulerpa verticillata*	no	4 (all negative)

Feeding acceptance of *E. timida* for different alga as potential chloroplast donors was tested. Feeding success was determined by the increase of photosynthetic yield in PAM-measurements.

**Figure 5:**
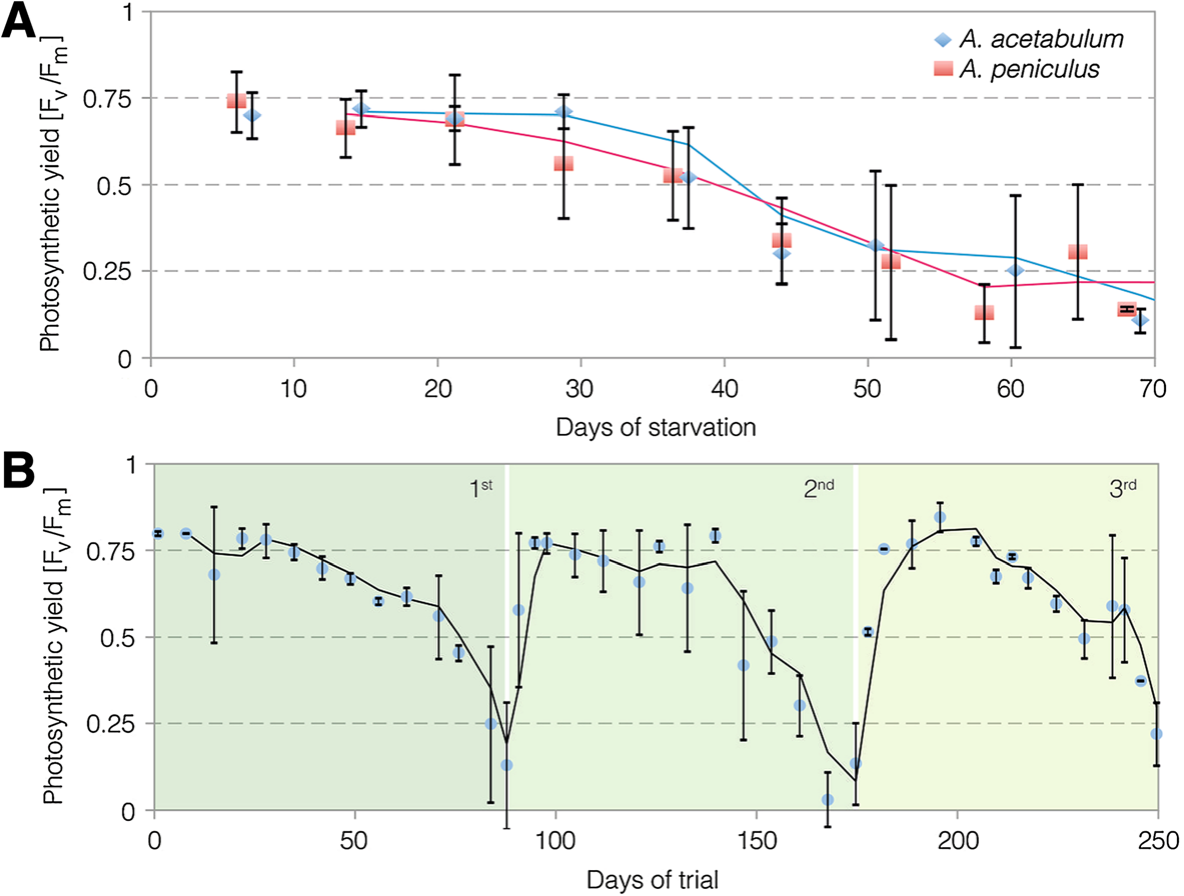
**Photosynthetic long-term capacities of *Acetabularia acetabulum *and *A. peniculus *chloroplasts in *Elysia timida *during starvation. A)** Long-term retention of chloroplasts from *A. acetabulum* and *A. peniculus* in *E. timida* during the second starvation phase. *E. timida* individuals had gone through a first starvation phase until depletion of former chloroplasts, than fed again for a month with either *A. acetabulum* or *A. peniculus* and consecutively measured for long-term retention during a second starvation phase with PAM fluorometry. Measurement days have been grouped. **B)** Long-term retention of chloroplasts from *A. acetabulum* and *A. peniculus* in an *E. timida* individual, which completed three starvation phases. First starvation reflects yields of chloroplasts from *A. acetabulum*, the second starvation phase those from *A. peniculus* and the third starvation phase again those from plastids of *A. acetabulum*. The curves of the second and third phase include the respective re-feeding phase of a month and the consecutive starvation phase. Means and standard deviations are calculated for three consecutive measurements per measurement day.

All surviving individuals from phase 2 with a feeding phase of one month were subjected to a second starvation period. Out of the nine individuals from each alga, five (on *A. acetabulum*) and four (on *A. peniculus*) completed this second starvation phase (phase 3) until yield values again approached zero. Also the course of the photosynthetic capacities during the subsequent starvation phase (phase 3) revealed similar retention characteristics. Yield values started to decrease slightly sooner in chloroplasts from *A. peniculus* on average, but individuals from both algal donor groups revealed high photosynthetic activity throughout approximately one month and subsequent parallel degradation (Figure 5A).

One individual lived for so long that after a first starving phase (phase 1) and new feeding of *A. peniculus* (phase 2 and subsequent phase 3), it could go through an additional feeding phase with *A. acetabulum* (Figure 5B). As it survived more than 8 months in the experiment — and had also grown up for some time previously in the laboratory system — it had reached an age of approximately ten months and was thus the individual of *E. timida* with the longest recorded life span in the laboratory so far. Comparing the two curves of photosynthetic activity during and after re-feeding with each algal plastid donor, demonstrates that photosynthetic capacities were very similar for chloroplasts of the two different algae species within the same individual (Figure 5B). PAM Yield values during the feeding phase were slightly higher after the second re-feeding with *A. acetabulum* with 0.78 ± 0.04 compared to 0.70 ± 0.07 after the first re-feeding with *A. peniculus*. The duration of chloroplast retention after the stop of feeding was very similar for the two different algal donors in this particular individual: Yield values approached zero after 47 days and 49 days of starvation after being fed with *A. peniculus* and *A. acetabulum,* respectively.

## Discussion

*Elysia timida* individuals kept together in basins were observed to mate in their species-specific mating habit as previously described [33]. Furthermore, the typical orange coloration that egg masses of *E. timida* display due to extra-capsular yolk [15], as well as the size and relative stability of egg masses, is advantageous for culturing. Egg masses can be recognized easily in the basins and deposited and handled in separate containers, such as glass bowls. Marín and Ros [15] reported two seasonally varying development types for *E. timida* with intracapsular development into crawling juveniles during a short winter period, and lecithotrophic development with a short veliger phase of 3–4 days between hatching and metamorphosis in spring and autumn (with June to August missing) from Mazarrón Bay, Spain. Our observations from the first collections in Banyuls-sur-mer, France, in May/June 2010 also showed lecithotropic development with hatching of free-swimming veliger and crawling juveniles with a shell, which is cast off within 3–4 days. Single juveniles without a shell were rarely observed.

The development period from clutch deposition until hatching was longer in Banyuls-sur-mer (21 days) than in laboratory culture (17 days), which corresponded to the 16–18 days described by Marín and Ros [15]. This might have been influenced by temperature differences during clutch maturation, which was lower in Banyuls-sur-mer (16–22°C) than in the laboratory (20–23°C), again corresponding to the 20–24°C from Marín and Ros [15]. The lecithotrophic or intracapsular development with a metamorphosis induced without an external (algal) trigger is advantageous for culturing, since planktotrophic development imposes many more problems. It complicates water exchange due to free-swimming larvae and planktonic algae have to be provided, which implies additional effort and further possible difficulties. This was shown by Trowbridge [34] for *E. viridis*, and by Rumpho and coworkers [3,17] for *E. chlorotica*. In the latter species metamorphosis depends on the presence of filaments of the food algae for adult *E. chlorotica*, the heterokontophyte *Vaucheria litorea*.

The number of eggs in individual clutches in the laboratory culture system showed a slightly wider range (minimum of 19 to a maximum of 249) than reported by Marín & Ros [15] with 34–168 eggs per clutch, but ranges around a similar level. They counted 140 eggs per clutch on average, versus 108 in our laboratory system.

The close relationship between adult *E. timida* and its food algae *A. acetabulum* (Linnaeus) Silva, 1952 [35] was described recently [36]. Consistently, *E. timida* in laboratory culture accepted *A. acetabulum* and in our hands, juvenile *E. timida* were not observed to feed on *C. dalmatica* and *C. rupestris* purchased from a commercial supplier or other *Cladophora spp.* collected from the slugs’ natural habitat. Instead, juvenile *E. timida* fed directly on *A. acetabulum*, if young non-calcified stalks of the algae were provided. By contrast, Marín and Ros reported that juvenile *E. timida* fed on *C. dalmatica* before switching to *A. acetabulum* as an adult diet [15]. Giménez-Casalduero et al. [37] reported several cases of variations in *E. timida*, differing in morphological, reproductive or other features, including genetic differentiations. Molecular markers will be needed to analyze whether feeding differences suggest incipient speciation in *E. timida* or not.

As can be seen in the video of juvenile *E. timida* feeding on young stalks of *A. acetabulum* (Additional file 1), the juveniles are first transparent and only become green upon incorporation of the first chloroplasts from *A. acetabulum*. The chloroplasts integrated into the digestive gland cells differed clearly from those of *Cladophora dalmatica* (Kützing 1843) [38] chloroplasts documented in the literature for juvenile *E. timida*, which had a distinct pyrenoid [15]. Only 2–3 hours after the first initiation of feeding, plastid degradation had already commenced as shown by our tissue fixed 2-3 hours after feeding start. This shows that chloroplasts ingested intact, are then quickly digested; most likely, as juveniles need a large and rapid nutrient supply for their growth and development. Marín and Ros [15] reported that “host membranes of the phagocytic vacuole” (p. 98) surrounded chloroplasts from *C. dalmatica* in juvenile *E. timida.* A distinct, complete phagocytic membrane around chloroplasts from *A. acetabulum* could not be clearly defined in our electron micrographs of juvenile *E. timida*, but fragments resembling phagosome membranes were recognizable. In some cases, chloroplasts seem to lie freely in the cytoplasm with direct contact to the cytosol, in others, however, a distant gap between chloroplast and cytoplasm was observed, resembling the gap around chloroplasts of *C. dalmatica* in juvenile *E. timida* displayed by Marín and Ros [15]. Around those gaps, and especially around aggregations of several chloroplasts, parts of an enclosing phagocytic membrane can be seen in our electron micrographs, pointing to a possible correlation between degradation (digestion) and the presence of a phagocytic membrane – which however has to be backed up by more investigations.

Evertsen et al. [11] described phagosome membranes around chloroplasts in the sea slug *Placida dendritica* (Alder und Hancock, 1843) [39], which underwent degradation, while intact chloroplasts in *Elysia viridis* (Montagu, 1804) [40] lie directly in the cytoplasm. This corresponds to the report from Marín and Ros [15] of phagocytic membranes around the chloroplasts that were probably about to be degraded in juvenile *E. timida*, in contrast to intact chloroplasts without an additional layer of phagocytic membranes in adult *E. timida*. Our assumption that chloroplasts of *A. acetabulum* in juvenile *E. timida* are first digested after their initial intake is also in accordance with new findings of Pelletrau et al. [17]: chloroplasts in juvenile *E. chlorotica* are first degraded and an initial feeding phase of a week was needed until degradation decreased and chloroplast incorporation was established. Juvenile *E. timida* in our laboratory culture system died within 2–3 weeks after metamorphosis, when no feeding occurred. As kleptoplasts in photosynthetic sea slugs are not inherited through the eggs, a new repertoire of kleptoplasts needs to be established by every generation [3,17].

PAM fluorometry is an established method to measure photosynthetic capacities of long-term functionality of incorporated chloroplasts in sea slugs [4-6,11,30,41-43]. The data presented here indicate that *E. timida* maintains chloroplasts from *A. acetabulum* as well as from *A. peniculus* (R. Brown ex Turner) Solms-Laubach 1895 [44] in a functional state under laboratory conditions. Giménez-Casalduero and coworkers reported that *E. timida* fed on other algal species during laboratory trials, but unfortunately they did not specify on which [37].

Measurements of photosynthetic activity during the first phase of laboratory culture correspond well to earlier data from *E. timida* specimens that were measured for three weeks after collection from the natural habitat [4]. However, our data indicate better long-term capacities when compared to other published PAM-data of *E. timida*[5,30]. Jesus and coworkers [30] showed data for complete starvation phases until approaching F_v_/F_m_ values of zero for *E. timida* from Mar Menor and Mazarrón (Spain), but these individuals approached F_v_/F_m_ values of zero after about 40 days of starvation in contrast to the roughly 90 days on average in our experiments. They kept the slugs under lower light conditions of 40 μmol quanta m^-2^ s^-1^ in 10 hours light per day in contrast to our 86 μmol quanta m^-2^ s^-1^ light for 12 hours a day in the experimental setting.

The three serial trial groups showed differences in photosynthetic capacities indicating a potential influence of the different temperature conditions the individuals were exposed to, and which reflected seasonal influences. The instantaneous temperature optimum for carbon fixation in *E. timida* from Mar Menor, Spain, is described to be 25°C [45]. However, differences in the temperature optimum can be attributed to geographic variation. Clark et al. [46] also reported a temperature optimum of 25°C for carbon fixation in the chloroplasts of the sea slug *Costasiella ocellifera* (Simroth, 1895) [47]. This species is exposed to higher temperatures in its natural habitat in Florida than *E. timida* from the coldest part of the Mediterranean, namely Southern France. In contrast, Stirts and Clark stated an optimum of 15°C for maximum photosynthesis-based carbon fixation for *Elysia tuca* (Marcus and Marcus, 1967) [48], another chloroplast-equipped sea slug from Florida [49]. Further experiments focusing on the effect of temperature differences on long-term photosynthetic activity are however required to better understand the influence.

We were able to compare photosynthetic capacities of chloroplasts from two different algal donors in one and the same sea slug species. Long-term retention capacities with chloroplasts from both algal donors were very similar, showing that *E. timida* is able to store chloroplasts from an algal species that is – to our knowledge – not present in its natural habitat. How kleptoplasts stay photosynthetically active and are maintained in sea slugs over prolonged periods of time is still unresolved and remains the most intriguing aspect of the slug-kleptoplast association. Characteristics of the plastids alone cannot be the determining factor, as chloroplasts of the same food alga show different fates in different sea slug species. Such is the case for the alga *Codium fragile* (Hariot, 1889) [50], which serves as a food source for *Placida dendritica* that digests directly, as well as for *Elysia viridis* with short- to long-term retention of the kleptoplasts [11]. Christa et al. [7] showed that in the long-term retention sea slug *Plakobranchus ocellatus* van Hasselt, 1824 [51] only chloroplasts from one algal species (out of the originally 6 present) were likely contributing to photosynthesis after 64 days of starvation. This indicates differences in plastid characteristics across different algae. Retention of functional kleptoplasts of both algal donors in our study was shorter in the phases after renewed feeding than in the first starving phase after being taken from the culture system with constant feeding, possibly due to the advanced age of these animals and/or due to exhaustion from a complete depletion of plastids in the first phase of our experiments.

## Conclusions

We have been able to maintain populations of *Elysia timida* in continuous culture since June 2010. The finding that juvenile *E. timida* fed directly on the adult diet *A. acetabulum* is eminent in the light of future analyses, since only a single food source system has to be provided during complete life cycles. We also demonstrated for the first time that *E. timida* is able to perform long-term retention in culture with an alternative algal chloroplast donor. Transmission electron microscopy on juvenile *E. timida* showed that chloroplasts from *A. acetabulum* are first taken up intact while feeding for the first time, but degradation processes already commence 2–3 hours after initial uptake of algal material. This is clear evidence that juveniles need to feed and digest, before long term incorporation is possible. Intact chloroplasts appeared to reside directly in the cytoplasm, whereas gaps and membranous fragments, maybe of phagosomal origin, were observed around single or conglomerations of chloroplasts in various states of degradation. In conclusion, *Elysia timida* proved to be a tractable laboratory culture model system, which opens up new possibilities to investigate long-term plastid retention.

## Materials and methods

### Preliminary investigations for the suitability of the model organism

Initial investigations were performed at the marine biological institute Observatoire Océanologique at Banyuls-sur-mer (OOB), France, from April to June 2010. The first generation of *E. timida* individuals (n = 179) for the laboratory culture was collected in depths of up to 2 m in proximity to the OOB, along with different algae from their natural environment, including *A. acetabulum*. Slugs and algae were kept in 5 1 tanks with supply of seawater from the institutional circulation system of the OOB. The aquaria were regularly checked for freshly laid clutches, which were carefully removed and transferred into petri dishes. As soon as veliger larvae had metamorphosed into crawling juveniles, feeding trials were performed including young non-calcified stalks of *A. acetabularia* as well as *Cladophora spp.* collected in the direct environment of the slugs, as *C. dalmatica* was formerly reported as a food source for juvenile *E. timida*[15].

### Laboratory culture system

At the Institute for Molecular Evolution (IME), Heinrich-Heine-University of Düsseldorf, Germany, adult *E. timida* individuals were kept in 12 1 tanks in aerated artificial seawater (37–38 g/l, hw_Marinemix professional, hw-Wiegandt GmbH) in a climate chamber at 14–16°C. The water was partly renewed once a week, and in the meantime, evaporating water was replaced with demineralized freshwater from the laboratory system in order to keep a constant salinity level. Each tank was equipped with an aquarium pump to have a consistent water circulation and aeration. The influx area of the pumps was covered with filter cartridges to prevent slugs from streaming in and net barriers were installed to avoid getting slugs close to the pump. To provide free access to food algae, *A. acetabulum* was offered in the tanks in glass bowls covered by nets through which *E. timida* individuals could easily enter and exit but algae were kept in and prevented from floating. *A. acetabulum* was renewed when sucked out or looking old. The slugs were held under a light regime of 12 h to 12 h light/dark photoperiod in relatively low light intensities (tanks half-shaded with paper) of about 20–50 μmol quanta m^-2^ s^-1^ (PAR: photosynthetic active radiation, measured in water above bottom of tanks where individuals were located) (neon tubes Osram L 58 W/840 LumiLux cool white). This resembles holding conditions recently reported by Pelletreau et al. [17] for their cultivation of *E. chlorotica* at 12:12 L:D cycle at 40 μmol quanta m^-2^ s^-1^ (measured at light-water interface). As they stated for *E. chlorotica* – and as is as well accurate for *E. timida* – optimal light intensities for maintenance of specimens have not yet been experimentally verified. The applied light regime for *E. timida* in our culture system ranged around the value of 31.33 μmol quanta m^-2^ s^-1^ reported by Giménez-Casalduero and Muniain [52] for rapid saturation of the photosynthetic apparatus of *E. timida* from the Mar Menor lagoon. In our laboratory conditions, *E. timida* individuals appeared to be in good condition and did not reveal signs of light stress.

Freshly laid clutches were carefully removed and transferred into glass bowls and kept either in the climate chamber (14–16°C) or at room temperature (~19-24°C according to season) and under a light intensity of about 20–30 μmol quanta m^-2^ s^-1^ (PAR, measured above the containers). The artificial seawater for the cultivation of the clutches was filtered with a sterile-filtering-apparatus (142 mm Edelstahl-Druckfiltrationsgerät, Sartorius Stedim Biotech GmbH, Germany). The water in the glass bowls was regularly exchanged in part until veliger larvae or juveniles hatched. When hatchlings had reached a crawling juvenile state, they were provided with young stalks of *A. acetabulum* and kept in the small containers until they had grown to an adult state and could be placed into a 12 1 tank.

The algae were also cultivated in the climate chamber (14–16°C) or in the laboratory room at room temperature (~19-24°C according to season) and additionally in climate boxes at 21°C, all with 12 h to 12 h day/light regime (neon cultivation tubes, approximately 130–200 quanta m^-2^ s^-1^). For the medium of the algae, the artificial seawater (37–38 g/l, Tropic Marine Pro Reef, Zoo Zajac, Duisburg, or equivalent) was first filtered with a sterile-filtering-apparatus (142 mm Edelstahl-Druckfiltrationsgerät, Sartorius Stedim Biotech GmbH, Germany) and than enriched with f/2 medium (Guillards F/2 Marine Water, Sigma, 20 ml/l). Stock cultures of *A. acetabulum* (Mediterranean) and *A. peniculus* (Indopacific) were maintained according to Berger & Kaever [53].

### Feeding experiments with juvenile *E. timida*

In preliminary feeding experiments with juvenile *E. timida* at the marine laboratory OOB, the selected algae were added to petri dishes with clutches from which juveniles were hatching and the direct reaction of the juveniles was observed through a stereomicroscope (n ≥ 20 clutches). *A. acetabulum* and different *Cladophora spp.* that had been freshly collected from the sea in the surrounding area of *E. timida* populations were tested.

The feeding experiment with juvenile *E. timida* in the laboratory culture at the IME was performed in two series with offspring of two different clutches from the culture. Juveniles that had completed metamorphosis into the shell-less crawling juvenile form were carefully pipetted with a glass pipette (sterilized in boiling water) into four separate small glass bowls – 10 individuals per bowl from the first trial clutch, 15 individuals per bowl from the second trial clutch, respectively. In each of the four glass bowls one test algal species was added: *A. acetabulum*, *A. peniculus*, *C. dalmatica* or *C. rupestris*. The immediate reaction of the juveniles to the offered algae was observed through a stereomicroscope for more than 30 minutes per bowl. At this transparent state of the juveniles, feeding can clearly be determined by the intake of the green algal sap. Potential feeding progress was subsequently controlled every 2–3 days by recording green-colored versus transparent individuals for a period of 3 weeks. The feeding response to the laboratory-cultured *A. acetabulum* and *A. peniculus* was tested, and furthermore to *C. dalmatica,* which is described as a food source for juvenile *E. timida* in the literature [15]. *C. rupestris* was included as an additional *Cladophora*-species.

### Long-term retention PAM fluorometry experiment

The experiment to observe capacities of long-term retention of chloroplasts from different algae in *E. timida* was performed in the laboratory culture system at the Institute for Molecular Evolution, from January 2012 to October 2012. In total, 50 individuals of *E. timida* from the laboratory culture were included (one additional individual had been excluded directly from the analysis as it died after only 7 days of observation). The overall 50 individuals were divided into three serial groups, the first starting January 31th 2012 (n = 24, temperature range: 19(or lower)-22°C), the second starting March 13th 2012 (n = 16, temperature range: 20–22°C) and the third starting June 25th 2012 (n = 10, temperature range: 19–24°C). Regular interval temperature measurements started end of March to avoid overheating due to rising temperatures in spring and summer.

During the experiment, the slugs were kept individually in petri dishes under a 12 h to 12 h dark/light regime with light intensities of 86 μmol quanta m^-2^ s^-1^ (PAR, measured in air above petri dishes) provided by full spectrum lamps (Androv Medicals, Germany). Photosynthetic activity was measured as maximum quantum yield of fluorescence for photosystem II with a Pulse Amplitude Modulated Fluorometer (Photosynthesis Yield Analyzer Mini PAM, version 2.0, WALZ, Germany) following the methods after Wägele and Johnsen [43]. For the measurement, the fibre optic was held above the slug with a distance of 0,5 – 1 cm covering the body region with the parapodia well with the sensor of a cross section of 5 mm. Three consecutive measurements with the possibility to acclimate again in between were taken of each individual. As F_v_/F_m_ values decreased considerably during the course of starving periods, the sensitivity of the Mini-PAM was individually adapted by putting the parameter ‘outgain’ from level 2 (default) to higher levels, up to level 8. Ambient light conditions were measured with a light sensor connected to the Mini-PAM (US-SQS/L, Walz, Germany).

The experiment was performed in the following 3 phases:

Phase 1: Individuals grown on *A. acetabulum* were held separately without any further food supply until yield values approached 0, assuming that incorporated chloroplasts were degraded to a non-functional state. Phase 2: Individuals were than allowed to feed on the different newly provided test algae for a month in order to assure that they recovered completely from the starving period and could fully replenish with new chloroplasts to a state of storing. *A. acetabulum* and *A. peniculus* from the culture system were provided to compare two related species of which one is not the natural food due to separate geographic distribution. *Cladophora dalmatica*, described as a food source for juvenile *E. timida*[15], was also comparatively provided. Furthermore, *Vaucheria litorea* was offered as a comparative food alga (*V. litorea* K-0379, SCCAP (Scandinavian Culture Collection of Algae and Protozoa)), as it is described as chloroplast donor of *E. chlorotica* with extensive durations of long-term retention of chloroplasts [3,25-27]. Furthermore, *Caulerpa verticillata* (collected at the Mote Laboratory, Florida Keys, USA) was included because it was observed to be a potential chloroplast donor for long-term retention in *Elysia clarki* (unpublished results VS and HW). Feeding on the various algae was supervised by measuring photosynthetic activity. Phase 3: After the feeding phase of one month, the food algae were removed and the long-term retention photosynthetic performance of the individuals with the newly incorporated chloroplasts was surveyed by regular PAM fluorometry measurements. For the evaluation of PAM fluorometry data, means and standard deviations were calculated first for the three consecutive measurements per individual per day and then grouped for the respective group analyses.

### Transmission electron microscopy

For electron microscopic examinations of the very first incorporation of chloroplasts, juvenile *E. timida* were fixed in a mix of 2% glutaraldehyde and 2% paraformaldehyde in 0.1 M Cacodylate buffer after observed feeding on *A. acetabulum* in two time series: the first after 2–3 hours since the beginning of feeding and continued feeding until fixation; the second after 2 days since the beginning of feeding and continued free access to the food alga. The samples were post-fixed with 1% OsO_4_ and dehydrated in an ethanol series and finally embedded in Epon. Ultrathin sections were stained with uranyl acetate and lead citrate and observed at 80KV in the transmission electron microscope (Hitachi H7500) at the OOB.

### Availability of supporting data

The data sets supporting the results of this article are available by the responsible author (HW).

## Abbreviations

IME: Institute for molecular evolution; OOB: Observatoire Océanologique Banyuls-sur-mer; PAM: Pulse amplitude modulated; TEM: Transmission electron microscopy.

## Competing interests

The authors declare that they have no competing interest.

## Authors’ contributions

VS performed collections and preliminary investigations to start the laboratory culturing system, maintenance of the culture system, feeding experiments, video recordings and TEM investigations of juveniles, the long-term retention PAM-experiment, data processing and drafted the manuscript. KH participated in the design of the study, established protocols for the laboratory culturing system and partly supervised the establishing of the laboratory culture system by SG. SG performed collections and preliminary investigations to start the laboratory culture system, interpretation of data and established the culture methods at the laboratory. MLE performed TEM investigations of juveniles with VS and assisted in interpreting the results. DM established the culture of *A. acetabulum* and *A. peniculus* and provided specimens and protocols for culturing. WM and SBG initiated, designed and supervised the laboratory culturing system for the slugs and revised data interpretation and final analysis. HW initiated the study, collected material, designed experiments and helped in data interpretation and drafting the manuscript. All authors have revised the manuscript critically for important intellectual content and approved the final manuscript.

## Authors’ information

VS is PhD student under the supervision of HW and worked in a collaboration project with the culture system at the department of WM. KH was postdoctoral student at the Institut für Molekulare Evolution, Heinrich-Heine-Universität Düsseldorf. SG was diploma student at the Institut für Molekulare Evolution, Heinrich-Heine-Universität Düsseldorf. MLE is technical assistant for microscopy at the Observatoire Océanologique, Banyuls-sur-mer. DM is professor at the Institut für Zelluläre und Molekulare Botanik, Rheinische Friedrich-Wilhelms-Universität Bonn. SBG is postdoc in the group of WM, who is professor at the Institut für Molekulare Evolution, Heinrich-Heine-Universität Düsseldorf. HW is professor at the Zoologisches Forschungsmuseum Alexander Koenig, Bonn.

## Supplementary Material

Additional file 1: Video 1 Juvenile *Elysia timida* feeding. Metamorphosed into the shell-less juvenile state, young transparent *E. timida* feed for the first time – on young stalks of *Acetabularia acetabulum* – and turn green through the incorporated chloroplasts. Click here for file
